# The Role of the Endocannabinoid System in Human Gametogenesis

**DOI:** 10.3390/ijms26093996

**Published:** 2025-04-23

**Authors:** Nina Montik, Daniele Crescenzi, Carolina Marzocchini, Irene Lubinski, Linda Grementieri, Sonia Peruzzi, Marta Lombó, Andrea Ciavattini, Oliana Carnevali

**Affiliations:** 1 Gynecologic Section, Department of Odontostomatologic and Specialized Clinical Sciences, Università Politecnica delle Marche, 60123 Ancona, Italycarolina.marzocchini@ospedaliriuniti.marche.it (C.M.); irene.lubinski@ospedaliriuniti.marche.it (I.L.); sonia.peruzzi@ospedaliriuniti.marche.it (S.P.); a.ciavattini@staff.univpm.it (A.C.); 2 Department of Molecular Biology, Universidad de León, 24071 León, Spain; mloma@unileon.es; 3 Department of Life and Environmental Sciences, Polytechnic University of Marche, 60131 Ancona, Italy; o.carnevali@staff.univpm.it

**Keywords:** endocannabinoid system, human reproduction, gametogenesis

## Abstract

The endocannabinoid system (ECS) is a complex endocrine network involved in maintaining body homeostasis. It comprises endocannabinoids, their receptors (CB1 and CB2), and the enzymes and transporters responsible for their synthesis and degradation. While the ECS’s role in the nervous system is well established, its functions in other organs and peripheral tissues, including the cardiovascular, gastrointestinal, and reproductive systems, remain incompletely understood. With the increasing use of marijuana, particularly among individuals of reproductive age, concerns have emerged regarding its potential impact on male and female fertility. Phytocannabinoids (∆9tethrahydrocannabinol and cannabidiol), as well as synthetic cannabimimetic drugs, interact with the ECS, influencing sperm and oocyte physiology and reproductive outcomes. Recent research has identified ECS-related biomarkers with potential applications in infertility diagnosis, particularly in assessing male fertility with greater precision. Furthermore, emerging evidence suggests that ECS signaling pathways are involved in epigenetic modifications, which may influence health maintenance, disease susceptibility, and transgenerational inheritance patterns. These findings highlight the therapeutic potential of ECS modulation in reproductive disorders and broader medical applications. This narrative review aims to elucidate the role of the ECS in human reproduction, with a particular focus on the influence of endocannabinoids on gametogenesis. While current research underscores the critical role of the ECS in fertility, further investigations are needed to fully elucidate its underlying mechanisms and its broader implications for reproductive health and therapeutic interventions.

## 1. Introduction

The endocannabinoid system (ECS) is a complex endocrine system involved in maintaining the body’s homeostasis. The ECS comprises compounds naturally synthesized within the human body called endocannabinoids, their receptors, and the group of enzymes and transporters responsible for their synthesis and degradation [1].

The prefix endo- ahead of the word “cannabinoids” suggests that these compounds are similar in chemical structure and biological function to phytocannabinoids (PCs), which are the exogenous molecules present in recreational cannabis and responsible for the psychotropic effects of these drugs. ∆9tethrahydrocannabinol (THC) and cannabidiol (CBD) are the main phytocannabinoids [2].

Marijuana is indeed one of the most widely used recreational drugs, and its use during the reproductive age has raised concerns about its potential effects on female and male fertility. For example, THC and CBD use may negatively affect female fertility by disrupting ovulation, hormone balance, egg quality, and implantation processes [3]. The exposure to the cannabinoid compounds released by marijuana could also be a contributing cause to male infertility. Multiple in vivo and in vitro studies have explored the impact of marijuana on the hypothalamic–pituitary–gonadal axis, as well as its effects on spermatogenesis and key sperm functions such as capacitation, motility, and the acrosome reaction [3]. On the other hand, another important role and topic of debate is the use of marijuana as a medical drug. Proponents claim that it is an effective treatment for symptoms of patients with serious health diseases, among which are cancer-related pain and epilepsy. Nevertheless, opponents sustain that it has many unwanted side effects that negate the beneficial effects, and one specific area of concern is reproductive health [4].

Endocannabinoids are lipophilic bioactive molecules. To date, anandamide (AEA), also known as N-arachidonoylethanolamide, and 2-arachidonyl-glycerol (2-AG) are the best-known and best-studied compounds belonging to this family. AEA and 2-AG are synthesized from lipidic precursors of phospholipidic cell membranes [5,6,7]. The endocannabinoids act as endocrine, paracrine, and autocrine signaling messengers; they are synthesized and released in bloodstream, in the lymphatic system, and in extracellular space. Endocannabinoid signaling is regulated by a system of degrading enzymes that limit the action of the effector molecules. AEA is metabolized by the fatty acid amid hydrolase (FAAH) and 2-AG is degraded by monoacylglycerol lipase (MGLL) [1,8].

The main receptors of ECS are cannabinoid receptor type 1 (CB1) and cannabinoid receptor type 2 (CB2) [9]. The CB1 receptor was initially localized in CNS, and CB2 in peripheral tissues. However, CB1 has recently been found in several tissues, such as in the cardiovascular and reproductive systems and the gastrointestinal tract [10]. Conversely, CB2 receptors are widely distributed in the periphery, and particularly, in the immune system, but it has been recently found in CNS [11]. AEA binds both CB1 and CB2 receptors, but its affinity for CB1 is four-fold higher than for CB2 [12]. 2-AG shows a lower affinity for CB1 than AEA, but its concentration in CNS is typically 100–1000 times higher than AEA [4]. This difference in concentration is reflective of their distinct roles and the way they are synthesized and degraded within the CNS.

It has recently emerged that endocannabinoids bind also, although with a lower affinity, to the transient receptor potential cation channel subfamily V member 1 (TRPV1), also known as type 1 vanilloid receptor, that is involved in the nociceptive system and synaptic transmission [13]. Cannabinoid receptors and their enzymes have also been found in the reproductive system, both at the central and gonadal levels [14,15]. ECS is present in the human testis and in the ovary, where it plays a role that, to date, is not completely clear [16]. Two additional endocannabinoids, palmytoylethanolamide (PEA) and oleoylethanolamide (OEA), are part of the ECS. They do not act directly on CB1 and CB2 but enhance the biological activity of AEA and 2-AG by competing for hydrolysis with EC degradation enzymes and by binding TRPV1 receptors. Moreover, all the endocannabinoids have been shown to have a role in oxidative metabolism due to their protective action against reactive oxygen species (ROS) [17].

A more recently discovered and sophisticated mechanism of action of ECS is the modulation of gene expression through epigenetic changes. Epigenetic regulation of gene expression consists of chemical modification of DNA and histone tails, which leads to different chromatin architecture and accessibility to transcriptional factors. Methylation is the main chemical modification of DNA in epigenetic regulation. Through the covalent transfer of methyl groups (-CH3) in the gene promoter region, the chromatin structure is changed from an open (transcriptionally active) to a closed (transcriptionally inactive) state. Epigenetic modifications also comprehend non-coding RNAs, capable of interacting with every aspect of gene expression [18]. Moreover, gene-specific histone modifications have been associated with eCB signaling mediated by cannabinoid receptors in several tissues. ECS affects the function of the enzymes responsible for the addition and removal of functional groups (for example, methyl, acetate, and phosphate) to genome-associated proteins that control the transcriptional activity; in this way, the expression of genes is enhanced or reduced. The epigenetic regulation of gene expression has also been described in the reproductive system. For example, ECS plays a role in spermiogenesis by remodeling the chromatin of spermatids, affecting the maturation process [19]. Similarly, the ECS, by epigenetic changes, affects the oogenesis by acting on granulosa cells [20]. Interestingly, ECS activity is otherwise regulated through epigenetic changes in both physiological and pathological conditions; among the main effectors on ECS, diet, exercise, smoking, and drugs are mainly responsible for the downregulation of EC receptors and enzymes synthesis [19].

The study of phytocannabinoids and the development of synthetic cannabimimetic drugs have garnered significant attention toward the endogenous cannabinoid system (ECS), which mediates responses to THC and CBD. Consequently, scientific research on the ECS has expanded exponentially in recent years. While its role in the nervous system is well established, its functions in other organs and peripheral tissues remain incompletely understood [2].

This review aims to elucidate the role of the ECS in human reproduction, with a particular focus on the influence of endocannabinoids on gametogenesis.

This article distinguishes itself from previous works by focusing on the literature and data related to the ECS in human gametogenesis. Research on animals have been considered only when authors compared the effect of ECS on gametogenesis of humans and other non-human mammals.

## 2. ECS and Male Gametogenesis

Human spermatogenesis is a complex and highly regulated biological process leading to the production of mature sperm cells, known as spermatozoa, in the male reproductive system. Spermatogenesis is essential for the establishment and maintenance of male fertility; any disruptions or abnormalities in this process can lead to male infertility [21].

This 74-day-long process occurs in the human testis, specifically within the seminiferous tubules, and can be divided into three distinct phases: mitotic and meiotic divisions (spermatocytogenesis) and spermiogenesis [22].

The mitotic phase involves the proliferation and differentiation of spermatogonial stem cells (SSCs), which are undifferentiated cells in the basal membrane of seminiferous tubules. SSCs are capable of continuous self-renewal, maintaining a steady stem cell pool in the testes, but can also give rise to a progeny of spermatogonia at different stages of development until they complete their maturation into spermatocytes [23]. SSCs undergo differentiation into A-paired and then A-aligned spermatogonia by several mitotic divisions. In Aal spermatogonia, the expression of proliferating-associated genes commits the cells to progressively differentiate into A1, A2, A3, A4, and B spermatogonia, which finally generate preleptotene spermatocytes. Preleptotene spermatocytes are diploid cells (2n) and undergo two successive cell divisions called meiosis I and meiosis II. Meiosis I is a 16-day-long process and consists of different phases: prophase I, metaphase I, anaphase I, and telophase I. During prophase I, homologous chromosomes pair up and undergo crossing over, exchanging segments of homologous chromatids. The homologous chromosomes then separate and move to opposite poles of the cell, resulting in the formation of two haploid daughter cells (n), known as secondary spermatocytes. Each secondary spermatocyte undergoes meiosis II, resulting in the formation of four haploid round spermatids [21,23].

Spermiogenesis is the final stage of spermatogenesis and lasts about 26 days; in this phase, round spermatocytes undergo major morphological changes; in particular, the reorganization of the nucleus and cytoplasm, modification of the cell’s shape, the development of the acrosome, and the tail. At the end of the process, mature polarized spermatozoa are released in the lumen of the seminiferous tubules (spermiation) [23]. Spermatozoa are differentiated and highly specialized cells capable of active motility in order to swim through the mucus of the female genital tract and reach, bind, and penetrate the oocyte [24]. They have dimensions ranging from 50 to 70 μm in length, and their morphology is characterized by three different parts: head, midpiece, and tail [25].

The head is a smooth-contoured, oval-shaped structure which contains the haploid nucleus in the form of tightly compacted chromatin; the nucleus is covered for two-thirds of its anterior portion by a vesicle, called the acrosome, which contains various hydrolytic enzymes essential for breaking down the egg’s zona pellucida. The midpiece is the central region of the sperm cell and is densely packed with mitochondria, which provide energy for sperm mobility [25].

The tail, also known as flagellum, is an elongated structure apically inserted to the post-acrosomal end of the midpiece; it is composed of microtubules arranged in a 9+2 pattern, with nine outer microtubule doublets surrounding a central pair of microtubules. The tail is responsible for propelling the sperm forward through rhythmic, wave-like movements known as flagellar or tail beating [24]. The sperm cell is enclosed in the plasma membrane that provides structural integrity and contains proteins and receptors involved in sperm–egg interactions [24]. Throughout spermatogenesis, various hormonal, non-hormonal, and paracrine signals regulate the process. Follicle-stimulating hormones (FSHs) acting on Sertoli cells and testosterone produced by Leydig cells are two key hormones involved in stimulating and supporting spermatogenesis [26]. Sertoli cells within the seminiferous tubules support sperm cells’ maturation, producing important growth factors, such as androgen-binding proteins, ceruloplasmins, transferrins, Mullerian duct inhibiting substances, c-kit ligands, inhibins, and glial cell line-derived neurotrophic factors [22].

The enigmatic network of ECS plays a pivotal role in regulating various physiological processes, including fertility and reproduction. As mentioned previously, even if ECS is primarily associated with the nervous system and immune system, it also has important functions in the male reproductive system, including sperm production.

Research suggests that the ECS is involved in the regulation of sperm function, including sperm production, maturation, motility, and fertilization capacity. The presence of cannabinoid receptors, such as CB1 and CB2, has been identified in human sperm cells and in the human testis. These receptors interact with eCBs, such as AEA and 2-AG, as well as with exogenous cannabinoids, like THC. Recently, investigations have focused on the identification of ECS components in spermatozoa and on the association of their presence with specific parameters of sperm quality and function. The ECS receptors were found in human spermatozoa, however, with some discrepancies; while most studies demonstrated the presence of CB1 alone, the other canonical receptor, CB2, was also detected in sperm, although at a much lower expression level. Concerning the CB1 receptor, it binds extracellular AEA and is localized prevalently in the sperm membranes of the head and on the mitochondria; in addition, spermatozoa possess all the biochemical machinery necessary to synthesize (NAPE-PLD) and degrade (EMT and FAAH) AEA [7,25,27]. CB1 and NAPE-PLD have been detected in the post acrosomal region, in the midpiece, and in the tail region of spermatozoa, whereas the localization of an additional endocannabinoid receptor, the transient receptor potential cation channel subfamily V member 1 (TRPV1), also activated by intracellular AEA, has been restricted to the midpiece and tail region. CB2 is localized in the sperm head membrane [7,28].

In 2019, the study of Nielsen J.E. et al. was the first to demonstrate the presence of a peculiar pattern of the signaling pathway of ECS in various human testicular cell types, including germ cells at different stages of spermatogenesis [29]. Specifically, the researchers identified a germ cell maturation stage-specific pattern of the ECS expression, with scarcity of most of the ECS components in early spermatocytes, evident expression in pachytene spermatocytes, and the strongest aspect in the post-meiotic spermatids. This study revealed notable findings regarding the CB2 receptor, which was found to be moderately expressed in a subset of spermatogonia, absent in primary spermatocytes, and re-expressed in post-meiotic germ cells. It represents the first evidence of CB2 receptor presence in human spermatogonia. The expression pattern reported aligns with previous mouse studies highlighting the role of the endocannabinoid system (ECS) in regulating the transition from mitosis to meiosis. In brief, the authors showed that the shift from spermatogonia to early primary spermatocytes—marking the mitosis-to-meiosis transition—was associated with a downregulation of ECS receptors and related metabolic enzymes. Conversely, post-meiotic germ cells exhibited elevated levels of ECS catabolic enzymes, possibly to ensure efficient endocannabinoid turnover during spermiogenesis or to support their function in mature sperm outside the testes [29].

**Figure 1:**
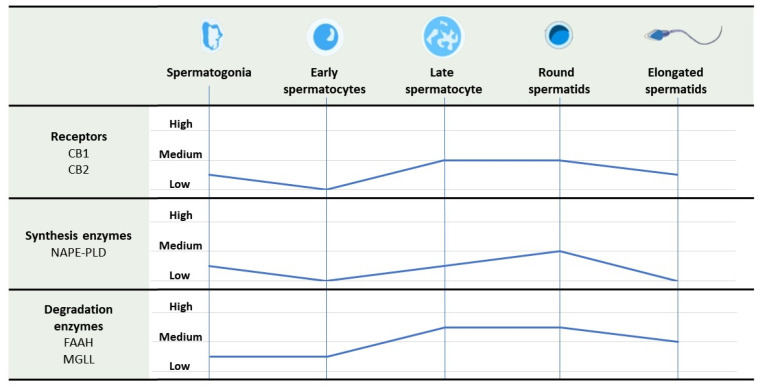
Levels of expression of receptors and synthesis and degradation enzymes of endocannabinoid system throughout the spermatogenesis. Abbreviations used: CB1: cannabinoid receptor type 1; CB2: cannabinoid receptor type 2; FAAH: fatty acid amide hydrolase; MGLL: monoacylglycerol lipase; NAPE-PLD: N-acyl phosphatidylethanolamine-specific phospholipase D.

Figure 1 shows the changes in expression of receptors and synthesis and degradation enzymes of the endocannabinoid system throughout the spermatogenesis.

Previous studies demonstrated that Sertoli cells express lower levels of the ECS components with the absence of the receptors. The presence of ECS in peritubular cells, including the expression of CB2, highlights the involvement of ECS in the testicular microenvironment beyond just germ cells [29]. Peritubular cells are essential for supporting and nourishing spermatogenic cells, and their interaction with ECS components suggests a role in regulating spermatogenesis. The balance between the synthesis and degradation of endocannabinoids within the seminiferous epithelium likely plays a critical role in regulating spermatogenesis. The observation of a relative silencing of ECS receptors and metabolizing enzymes during the transition from spermatogonia to early primary spermatocytes (i.e., from mitosis to meiosis) is intriguing. As previously mentioned, it suggests a dynamic regulation of ECS activity during different stages of spermatogenesis. This downregulation of ECS components could indicate a shift in regulatory mechanisms as germ cells progress through meiosis, reflecting the changing demands and processes of sperm cell development [29]. Overall, these findings underscore the intricate involvement of the ECS in regulating spermatogenesis and highlight its potential as a target for understanding and potentially treating male infertility and reproductive disorders.

Both receptors, CB1 and CB2, as well as the AEA synthesizing enzyme NAPE-PLD have been detected in the Leydig cells. In the interstitial compartment, a poor representation of the degrading enzymes ABHD2 and FAAH has been detected, possibly reflecting the participation of AEA in steroidogenesis. MGLL staining has been weakly detected in Leydig cells while highly present in blood vessel walls [29].

A consistent number of studies have shown that the activation of cannabinoid receptors in sperm cells can modulate various aspects of sperm function. For example, activation of CB1 has been associated with decreased sperm motility, which may impair the ability of sperm to reach and fertilize the egg [28]. Additionally, disruption of the ECS signaling in the testes can affect sperm production and quality [28,30]. From a functional point of view, the role of anandamide (AEA) in promoting the fertilizing ability of human sperm through the activation of TRPV1 receptors sheds light on the complex mechanisms underlying sperm maturation and fertilization. Significant levels of AEA are present in human seminal plasma, as well as in mid-cycle fallopian tubal fluid and follicular fluid. This supports the notion that human sperm are exposed to anandamide (AEA) in a sequential manner—first during their storage in the epididymis and later as they travel through the female reproductive tract—implying a regulatory function of the endocannabinoid system in sperm activity. Experimental studies conducted in vitro indicate that AEA, acting via the CB1 receptor, can influence key sperm processes such as motility, capacitation, and the acrosome reaction [31]. In particular, AEA promotes the sperm fertilizing ability by activating TRPV1 receptors. TRPV1 ion channels are crucial for sperm capacitation and the acrosome reaction, both of which are essential for successful fertilization. Moreover, mammalian sperm undergo capacitation, a process essential for their fertilizing ability. During capacitation, sperm motility changes, becoming hyperactive, which enables them to reach the oocyte. This process involves increased flagellar curvature and wider lateral head movements, providing the sperm with the necessary strength for penetration. Capacitation also involves a calcium influx, further facilitating the process. In addition, another critical aspect of capacitation is the sperm’s ability to further undergo the acrosome reaction. This reaction involves the release of enzymes from the acrosome, facilitating penetration through the zona pellucida of the oocyte. This step is crucial for successful fertilization. Finally, the regulation of the capacitated state is tightly associated with the sperm’s proximity to the oocyte. Premature initiation of capacitation can lead to spontaneous acrosome reaction, hindering fertilization and highlighting the importance of precise timing in the fertilization process [32].

An interesting study carried out in 2012 by Lewis et al. found a substantial modulation of AEA metabolism in sperm from infertile men [33]. In infertile sperm, both the synthesis of anandamide (AEA) via the NAPE-PLD enzyme and its breakdown by FAAH appear to be compromised, resulting in a marked decrease in AEA levels within the seminal plasma of these patients. The lack of detectable TRPV1 activity, coupled with the reduced AEA concentration, may contribute to diminished fertilization potential. AEA normally acts to prevent premature capacitation in newly ejaculated sperm, and the data suggest that this CB1-mediated signaling mechanism may be disrupted in cases of male infertility. The endocannabinoid system (ECS) is thought to help maintain sperm in a dormant, incapacitated state until contact with the oocyte occurs. Therefore, a reduction in AEA might cause sperm to exit this quiescent state too early, leading to untimely capacitation and acrosome reaction, ultimately impairing their ability to fertilize the egg. Furthermore, Lewis et al. [33] demonstrated for the first time that human sperm contain components involved in 2-AG metabolism, with infertile sperm showing a higher degradation rate and lower overall levels of 2-AG. [33]. For that reason, in infertile men, a decrease in 2-AG levels in seminal plasma may also reduce the fertilizing capacity of sperm through a not well understood mechanism [33]. Those results are consistent with another similar study which demonstrated that 2-AG affects the in vitro functionality of human sperm by lowering motility skills, inhibiting capacitation and triggering the acrosome reaction [31].

### Epigenetic Mechanisms and Male Gametogenesis

Disorders of the ECS have been associated with many pathological conditions including infertility, indicating that the modulation of this system may have a pivotal role for the maintenance of health status and disease handling. Lifestyle and environmental factors can influence gene expression through epigenetic mechanisms and this environmental-dependent modulation of gene expression usually occurs at transcriptional, post-transcriptional, and translational levels through modifications to DNA and histone proteins or the production of regulatory non-coding RNA. Recent research indicates that the ECS can be epigenetically regulated in biological tissues [18]. Additionally, substances such as endocannabinoids, phytocannabinoids, and cannabinoid receptor agonists and antagonists can induce epigenetic changes, either widespread or gene specific. These changes may have implications, not only for the individual but also for future generations, as they can be passed down through gametes, potentially leading to transgenerational epigenetic inheritance [19]. Latest evidence suggests that the endocannabinoid system is subject to epigenetic modulation by various factors such as alcohol, diet, stress, smoking, exercise, and drugs. These factors can impact the expression of genes related to the endocannabinoid system, particularly CNR1, which encodes CB1, and the enzyme FAAH. These alterations can lead to changes in endocannabinoid signaling or tone. The identified epigenetic mechanisms involve several processes, including changes in DNA methylation (both global and gene-specific), modifications to histone tails such as acetylation, deacetylation, or methylation, and the production of specific microRNAs (miRNAs) in various brain regions, peripheral tissues, and cell lines. Reproduction is a process highly sensitive to environmental factors like diet, stress, or endocrine disruptor exposure among others. The emerging research on the epigenetic modulation of the endocannabinoid system in male gonads is shedding light on its roles in spermatozoa and its implications for fertility and embryo development. This modulation likely involves alterations in DNA methylation patterns, histone modifications, and microRNA expression within the male reproductive system. These epigenetic changes could influence sperm quality, fertilization potential, and early embryonic development, ultimately affecting fertility outcomes and embryo viability [18].

The initial evidence of epigenetic modulation of the endocannabinoid system in the gonad revolves around the expression of FAAH1 in Sertoli cells. The survival of Sertoli cells is dependent on the tone of AEA as well as the activity of FSH and estradiol. The epigenetic regulation of FAAH1 expression in Sertoli cells suggests that alterations in DNA methylation, histone modifications, or microRNA expression may impact the levels of the FAAH1 enzyme, thereby affecting the breakdown of endocannabinoids like AEA within the testicular environment. This modulation could have implications for germ cell development, spermatogenesis, and ultimately, fertility [34]. The FAAH1 gene promoter contains an estrogen-responsive element (ERE), indicating its responsiveness to estrogen signaling. Specifically, in primary mouse Sertoli cells, it has been observed that FAAH1 expression is under the control of estradiol. However, the transcriptional activation of FAAH1 by estradiol is a complex process that involves multiple molecular players. In particular, the binding of estrogen receptor beta (ERβ) to proximal ERE sequences (such as ERE2/3) is necessary for estradiol-dependent transcriptional activation of FAAH1. Additionally, the lysine-specific demethylase 1 (LSD1) is implicated in this process. LSD1 likely facilitates the removal of methyl groups from histone H3 lysine 9 (H3K9), leading to chromatin remodeling. Moreover, estradiol-mediated transcriptional activation of FAAH1 also involves decreased DNA methylation at CpG sites within the proximal promoter region of the gene. This decrease in DNA methylation further enhances the accessibility of the promoter for transcriptional machinery. Taken together, these findings highlight the intricate interplay between histone modifications and DNA methylation in the regulation of FAAH1 expression in Sertoli cells [35].

Concerning Leydig cells, despite the solid connection between its activity and the ECS, at present, there is no evidence of the possible epigenetic modulation of ECS in Leydig cells. Little is known about the reproductive effects of paternal cannabis exposure. There have been evaluated associations between cannabis or THC exposure and altered DNA methylation in human sperm. A study has shown at least 6640 CpG sites whose methylation status was altered in conjunction with cannabis use in males of child-bearing age, compared to non-using cannabis males [36]. Epigenetic modifications to the genome play an essential role in regulating gene expression over the life course and have been reported as a potential mechanism underlying the heritable effects of pre-conception cannabis exposure. Currently, it is known that DNA methylation is erased and establishes a new DNA organization within each generation, first just after fertilization, then again in the developing embryo wherein the methylation present in the primordial germ cells is erased, and after sex specification, is reestablished in a sex-specific manner. The reprogramming of the methylome is a highly orchestrated yet delicate process that may be susceptible to disruption by both internal and external factors. Given that spermatogenesis is an ongoing process throughout a man’s reproductive lifespan, exposure to substances such as cannabis has the potential to affect the stability of sperm DNA methylation patterns, possibly influencing the transmission of these epigenetic modifications to future generations. Although it remains uncertain whether environmentally induced DNA methylation changes in human sperm can be inherited, emerging evidence raises important concerns. For instance, a study by Watson and colleagues [37] demonstrated that offspring of rats exposed to THC exhibited widespread alterations in DNA methylation compared to controls. These findings suggest that the epigenetic effects of THC exposure in parents may be passed down through the germline, potentially via methylation modifications in gametes that escape reprogramming after fertilization. Should such methylation changes persist in the zygote, they could dysregulate the expression of key developmental genes, potentially resulting in embryonic lethality or altered growth patterns. However, these interpretations remain hypothetical, and additional studies in human populations are crucial to clarify the extent to which such epigenetic alterations may be inherited across generations [36].

The possibility of transmission of epigenetic alterations associated with exposure to THC could also concern the female counterpart. There are links reported between prenatal cannabis exposure (PCE) and undesirable birth outcomes. It has been hypothesized that PCE delivers a “first hit” to the endogenous cannabinoid signaling system (ECSS), which is compromised in such a way that a “second hit” (i.e., postnatal environmental stressors) may precipitate the emergence of a specific phenotype. Therefore, epigenetic factors because of PCE in utero could potentially operate transgenerationally, so that descendants (even those non-affected by PCE) are more susceptible to a “second hit”, which may result in an associated phenotypic syndrome. Regarding this aspect, many studies are still needed to demonstrate the aforementioned hypotheses [38].

During postnatal testis development, additional reprogramming of epigenetic marks occurs in two time frames: at the entry in meiosis, a process requiring CB2 activity, and in post meiotic stages, notably under CB1 control [18]. In detail, during spermatogenesis, CB2 exerts a pivotal role in meiosis entry and its hyper- or hypo-stimulation disrupts the temporal dynamics of the spermatogenesis with possible epigenetic mechanisms. In fact, JWH-133 (3-(1′1′dimethylbutyl) 1-deoxy-8-THC), a selective CB2 agonist, stimulates the expression of the meiotic genes c-Kit and Stra8, by upregulating H3K4me3 and downregulating H3K9me2 levels in genomic regions flanking the transcription start site, resulting in an accelerated meiosis entry. Prolonged treatment with the CB2 agonist JWH-133 has been shown to expedite spermatogenesis, whereas administration of the selective CB2 antagonist AM630 results in its delay. These findings underscore the crucial role of balanced endocannabinoid signaling in maintaining normal spermatogenic processes and highlight the potential negative consequences of exogenous cannabinoid exposure on male reproductive health. Disruption or artificial modulation of the endogenous endocannabinoid system (ECS) may compromise gamete quality and alter epigenetic signatures within germ cells—both of which are essential for successful fertilization and healthy embryonic development. Although still under investigation, such alterations raise the possibility of heritable epigenetic changes being passed to subsequent generations. Nevertheless, since these studies have only been conducted on mice so far, any conclusions are still hypothetical [39,40].

Another study, also conducted on mice, showed a reduced DNA hydroxymethylation and an increased DNA methylation at paternally expressed genes *Peg10* and *Plagl1* in sperm from JWH-133-exposed males. In detail, *Peg10* is a gene implicated in placenta formation and embryonic growth and *Plagl1* is considered a master regulator of growth and development of the fetus/placenta. DNA hypermethylation of those genes was found conserved in placentas from JWH-133 exposed fathers. It highlights the role of CB2 overactivation in causing alterations of sperm DNA methylation that are inherited by the next generation with negative implications for offspring growth [41].

During spermiogenesis, chromatin remodeling and DNA packaging are therefore the main nuclear events, consisting of a double-step process that requires histone replacement, first by transition proteins (TP2 and TP1) and then by protamines (PRM1 and PRM2), a class of small basic proteins. Another recent analysis stated the possible role of eCBs in chromatin remodeling during spermiogenesis. Experiments carried out on male mice homozygous for a CB1-null mutation (*CNR1*^−/−^) observed that CB1 activation regulates chromatin remodeling of spermatids by either increasing TP2 levels or enhancing histone displacement. Indeed, deletion of the CNR1 gene has been shown to impair proper chromatin packaging in sperm, primarily by reducing TP2 mRNA levels and interfering with the replacement of histones by protamines. This disruption leads to defects in chromatin condensation and compromises DNA integrity, as evidenced by elongated nuclear morphology in affected spermatozoa. Once again, the observations above highlight the potential risk to spermatozoa epigenome integrity following marijuana use and suggest that eCBs may act as epigenetic factors [19].

Considering the extensive involvement of the endocannabinoid system (ECS) in numerous physiological processes, it is plausible that environmental influences could epigenetically alter ECS function, potentially leading to impairments in reproductive health or modifications in gene expression that may be heritable across generations. Although the majority of current evidence stems from non-human experimental models, the limited number of human studies—often with small sample sizes and lacking offspring follow-up—restricts definitive conclusions. Nevertheless, the identification of similar DNA methylation patterns in both human and rodent sperm supports the relevance of animal data as a foundation for future human-focused research. Moreover, the therapeutic application of phytocannabinoids and synthetic cannabinoids raises concerns about the potential for transgenerational transmission of epigenetic alterations, emphasizing the importance of cautious evaluation in clinical and research settings [18].

In conclusion, male factor infertility is a significant issue, contributing to about 40% of cases where couples experience difficulty conceiving. This may depend on a reduced number of sperm due to reduced spermatogenesis or irregular maturation, or it may be caused by sperm dysfunction from metabolic deregulation or oxidative stress. Recent studies have demonstrated that the endocannabinoid system has a key role in the multifaceted process of male reproduction. Furthermore, exposure to exogenous cannabinoids, such as THC from cannabis, can interfere with the normal functioning of the ECS in sperm cells. Chronic cannabis use has been linked to a decreased sperm count, reduced sperm motility, and altered sperm morphology, potentially leading to male infertility. Investigating the role of the endocannabinoid system in male reproduction could provide valuable insights into the mechanisms underlying male infertility. Characterizing the expression and function of cannabinoid receptors and endocannabinoids in sperm cells could provide a better understanding on their involvement in sperm production, maturation, and function. Additionally, exploring how ECS signaling pathways interact with other regulatory systems in the male reproductive tract, such as hormonal pathways and oxidative stress responses, could provide a more comprehensive understanding of male fertility [42]. The present identification of the ECS as a family of new biomarkers to determine male infertility with more accuracy has enormous potential in fertility clinics. Overall, while the ECS undoubtedly plays a role in sperm physiology and male fertility, further research is needed to fully understand the mechanisms involved and the potential implications for reproductive health. Lastly, understanding the epigenetic mechanisms within the ECS could have significant implications for health maintenance and disease treatment, suggesting that modulation of the endocannabinoid system may be a promising avenue for therapeutic interventions in various disorders.

## 3. ECS and Female Gametogenesis

Oogenesis is the process during which the formation of the oocyte takes place. In human female PGCs, the transition from mitosis to meiosis begins from the fetal stage. In mammals, oogenesis can be divided into three phases. The first one (Phase I) is characterized by the specification of PGCs and their differentiation into oogonia. During the second one (Phase II), oogonia entrance into meiosis I (MI) occurs, followed by their differentiation into primary oocytes arresting at the diplotene stage of prophase of MI. At this moment, oogonia and pregranulosa cells coexist. This complex undergoes morphological changes leading to the flattening of pregranulosa cells which surround the primary oocytes. When reaching the last stage of meiotic prophase I that occurs during the fetal period, primary oocytes stand in a quiescent state in the primordial follicle till puberty. After sexual maturity, the final stage (Phase III) of oogenesis begins and it represents the process of oocyte maturation. During this stage, folliculogenesis takes place, involving the development of primordial follicles into antral or fully mature follicles. Ovulation is triggered by a surge in lueinizing hormone (LH) released by the pituitary gland, which induces the resumption of meiosis and ultimately allows for fertilization. Following the LH surge, oocytes within the antral follicles initiate chromatin condensation and undergo germinal vesicle breakdown (GVBD). This is followed by the completion of meiosis I (MI). Typically, approximately 10 to 20 quiescent oocytes resume meiosis during each menstrual cycle; however, only a single oocyte reaches full maturation and is subsequently ovulated. The oocyte then enters meiosis II (MII) and arrests at metaphase, pending fertilization. The transition from the germinal vesicle (GV) to MII involves intricate nuclear and cytoplasmic modifications essential for successful oocyte development and maturation [43,44]. The transition from the germinal vesicle (GV) stage to metaphase II (MII) takes place within the follicle and relies on tightly regulated communication between the oocyte and surrounding granulosa cells. These granulosa cells, which are somatic cells encasing the oocyte, differentiate into two populations following antrum formation: mural granulosa cells, which line the follicle wall, and cumulus granulosa cells, which maintain direct contact with the oocyte through gap junctions. Together, the oocyte and cumulus cells form the cumulus–oocyte complex (COC), the unit that is released during ovulation in vivo. Successful oocyte maturation requires bidirectional signaling—both through direct gap junctions and through paracrine mechanisms—between the oocyte and granulosa cells. Numerous factors within the COC contribute to the regulation of this maturation process, with increasing attention being drawn to the role of cannabinoid compounds [45].

**Figure 2:**
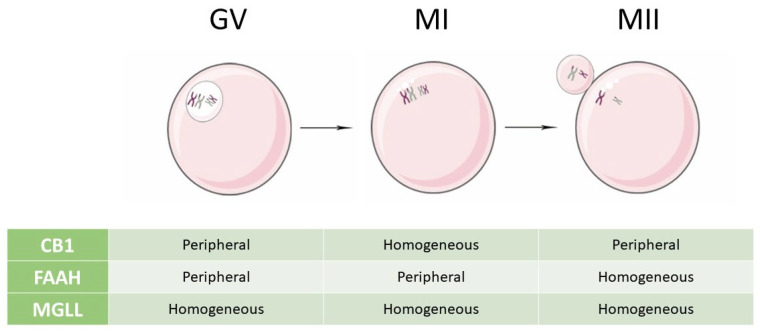
Pattern of expression of CB1 receptor, FAAH, and MGLL enzymes throughout oogenesis. The distribution of CB1, FAAH, and MGLL, studied with immunofluorescent analysis, varies through the different stages of maturation of oocytes. Abbreviations used: CB1: cannabinoid receptor type 1; FAAH: fatty acid amide hydrolase; GV: germinal vesicle; MGLL: monoacylglycerol lipase; MI: meiosis I; MII: meiosis II.

The ECS is known to impact the female reproductive system, where it affects folliculogenesis, oocyte maturation, and ovarian endocrine secretion. In addition, the ECS affects oviductal embryo transport, implantation, uterine decidualization, and placentation [46]. To demonstrate the implication of the endocannabinoid system in the process of oocyte maturation, some studies conducted on granulosa cells obtained from patients undergoing intracytoplasmic sperm injection (ICSI) confirmed the presence of the main receptors and enzymes responsible for endocannabinoid (AEA and 2-AG) synthesis and degradation also at the ovarian level. Different results were obtained regarding the concentration of endocannabinoid receptors and degrading enzymes. In particular, Agirregoitia et al. detected the presence of *CB1*, *FAAH*, and *MGLL*, but not *CB2* receptor transcripts. Analyzing the relative amount of each mRNA in the stages of oocyte maturation (GV, MI, MII), no significant differences were found [47]. In another study, focused on the investigation of the presence and differential distribution of *FAAH* and *MGLL* in relation to *CB1* during all stages of human oocyte maturation, only *FAAH* and *CB1* were found, but not *MGLL* transcripts [45]. In both studies, immunofluorescence was used to determine the precise localization of receptors and enzymes. Even though previous immunofluorescence analyses about *CB1, CB2, FAAH*, and *MGLL* did not observe any change in localization in human granulosa cells during nuclear maturation of oocytes, subsequent research has obtained different results, showing variations in the locations through the stages of the resumption of meiosis in human oocytes [48]. The presence of cannabinoid-degrading enzymes FAAH and MGLL in human oocytes was confirmed. Regarding FAAH, it was found peripherally at GV and MI stages, whereas at MII, it appears homogeneously present over the entire oocyte. On the other hand, MGLL was found homogeneously in oocytes from the GV to MII stages [45,47]. Comparing the immunocytochemical localizations of the FAAH and CB1 receptors, they are the same in the GV and MI stages (in the oolemma), although CB1 was also observed in the cytoplasm at MI. In stage MII, instead, FAAH results homogeneously present in the entire oocyte differently from CB1 that remains only in the oolema [45]. Figure 2 shows the n of expression of CB1 receptor, FAAH, and MGLL enzymes throughout oogenesis.

The observed shift in localization patterns of CB1 and FAAH during oocyte maturation suggests a coordinated redistribution aimed at modulating the local endocannabinoid environment as meiosis resumes. It is likely that CB1 is functionally active at the germinal vesicle (GV) stage when positioned at the plasma membrane, and is subsequently internalized as the oocyte progresses to the metaphase I (MI) stage. Given that FAAH is found in the same subcellular regions as CB1 during both GV and MI stages, it may play a role in degrading anandamide (AEA) that remains bound to CB1, particularly during the receptor’s internalization at the MI stage. According to the classical theory of G-protein-coupled receptor (GPCR) functionality, they have to reach the cell surface to act. After agonist binding, GPCR undergo a rapid desensitization and the ligand–receptor complex is internalized prior to being recycled back to the cell surface or being degraded. Therefore, it could be postulated that CB1 could be bound at GV stage (when it is localized at the plasma membrane) and subsequently internalized when the oocyte reaches the MI stage (when the CB1 receptor is localized within the cytoplasm). Then, the receptor could be recycled or degraded, being localized at the plasma membrane once again when the oocyte is blocked at the MII stage [49].

Additionally, at least at the GV and MI stages, FAAH might degrade free eCBs, which would enter the active site via the membrane. Then, the receptor could be recycled, being mostly localized at the plasma membrane once again when the oocyte is blocked at the MII stage, but FAAH cannot act at the periphery of oocyte because of its internalization [45]. The presence of MGLL, responsible for 2-AG degradation, one of the major metabolites of EC, in the cytoplasm of oocytes might help FAAH in ECS degradation [45].

In the context of the human female reproductive system, studies have shown that anandamide (AEA) levels increase in follicular fluid during oocyte maturation and reach even higher concentrations in the oviduct [49,50]. This observation supports the hypothesis that the endocannabinoid system (ECS) may play a regulatory role in the resumption of meiosis within the oviduct. On the other hand, high intracellular levels of cyclic AMP (cAMP), generated by adenylyl cyclase, are essential for maintaining meiotic arrest in oocytes. It is believed that this cAMP is primarily derived from cumulus cells and transferred to the oocyte through gap junctions [50,51]. Based on these findings, it is plausible to propose that the activation of CB1 receptors in granulosa cells—initially within the follicle and subsequently in the oviduct—could initiate intracellular signaling via Gi/o proteins, ultimately suppressing adenylyl cyclase activity and reducing cAMP synthesis, thereby promoting meiotic progression [45,52]. Ernst et al., with a study based on the characterization of the ECS in the human granulosa cell line KGN, a model commonly used for human granulosa cells, and its impact on gonadotropin sensitivity and steroid hormone synthesis under basal and FSH stimulated conditions, demonstrated a modulating role of the intrinsic ovarian ECS in the regulation of estradiol synthesis [53]. As proof of the above, a significant decrease in the basal estradiol level was caused by selective agonists of both cannabinoid receptors and inverse agonists/antagonists alone or in combination had no effect on basal estradiol level. Under FSH stimulation, cannabinoid effects were no longer visible. The decrease in estradiol was accompanied by the downregulation of *CYP19* (enzyme responsible for a key step in estrogen biosynthesis) while FSH receptor (*FSHR*) transcription was not. Moreover, treatment with cannabinoid receptor inverse agonists or antagonists resulted in a reduction of basal FSHR and CYP19 gene expression, without significantly affecting estradiol levels. This suggests that cannabinoids may engage alternative intracellular signaling mechanisms to modulate estradiol synthesis. These findings imply that the endocannabinoid system (ECS) can interact with gonadotropin receptors independently of follicle-stimulating hormone (FSH) signaling. Agonists targeting both CB1 and CB2 receptors similarly reduced basal estradiol production, yet had no effect on estradiol levels when stimulated by FSH. Notably, progesterone production remained unaffected by ECS modulation. Collectively, these results point to a regulatory role of the ovarian ECS in modulating steroidogenic activity within granulosa cells [53].

Regarding the role of ECS in female fertility, Costa et al. demonstrated AEA to be an important biomarker. Their study was conducted on human primary luteinized granulosa cells (hGCs) obtained from patients undergoing oocyte retrieval during assisted reproductive treatments [54]. It showed a decrease in cell viability in a concentration and time dependent manner when granulosa cells were exposed to elevated levels of AEA. Cells were also pre-incubated with CB1 (AM281) or CB2 (AM630) specific antagonists to determine which receptor might mediate the AEA effect. An interesting aspect that emerged from this study is that neither CB1 or CB2 antagonists were able to inhibit the decrease in granulosa cell viability. In addition, morphological studies of hGCs revealed an increase in cells with chromatin condensation and a mild reduction in cell density. AEA also induced a significant reduction in mitochondrial membrane potential (ψm). Due to morphological alterations associated with a decrease in Δψm, the analysis of the possible apoptotic cell death mechanism showed caspases activity involvement, suggesting an interplay between the intrinsic and extrinsic apoptotic pathways in AEA-induced cell death [54,55]. Although the physiological levels of anandamide (AEA) in follicular fluid are typically in the nanomolar range—lower than the concentrations used in some experimental settings—disruptions in AEA levels, particularly those resulting from the intake of exogenous cannabinoids, may lead to dysregulation of the endocannabinoid system (ECS). This imbalance could impair granulosa cell (GC) function and, consequently, female reproductive health. Endocannabinoid signaling is involved in nearly every phase of female reproduction, from oocyte maturation to childbirth, primarily through the activation of CB1, CB2, and TRPV1 receptors. Additionally, the synthesis and degradation of endocannabinoids by specific metabolic enzymes play a crucial role in maintaining the appropriate endogenous eCB tone required for reproductive processes. Many studies have shown that CB1 and CB2 are expressed in the preimplantation embryos, and that their activation by uterine AEA can interfere with embryo development. The gradient of AEA concentration is involved in controlling the oviductal transport of fertilized eggs and its development to the morula stage. This gradient is maintained by an increasing NAPE-PLD and decreasing FAAH expression in epithelial cells of the oviduct [56], associated with an increased expression of FAAH in embryonic cells which acts as protection against cytotoxicity of AEA [57].

It is also known that optimally balanced eCB signaling is critical to synchronize preimplantation embryo development and prepare the endometrium for implantation [56], demonstrated by the detected high levels of AEA and CB1 in non-receptive uteri and inter-implantation sites. This supports the view that a ‘metabolic control’ of eCB tone has a critical impact on uterine receptiveness [58,59].

**Figure 3:**
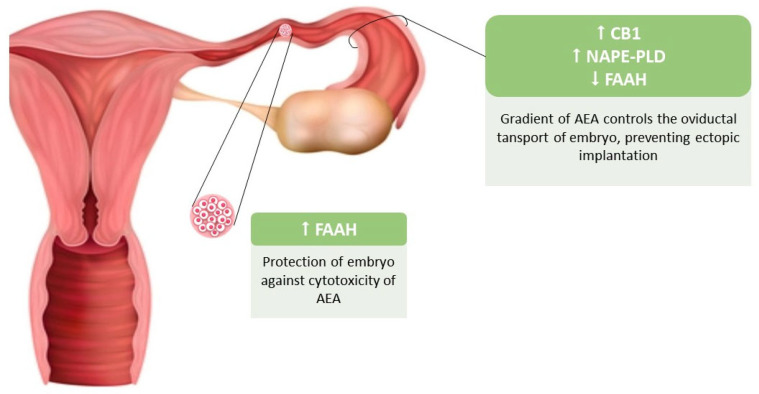
Expression of CB1 receptor, FAAH, and NAPE-PLD enzymes during preimplantation embryo development and migration through the fallopian tube. The high expression of CB1 receptors and NAPE-PLD enzyme in the oviduct, corresponding to a high endocannabinoid tone, protects against the ectopic implantation of the embryo during development and migration to the uterine cavity. The high expression of FAAH enzyme by the embryo counteracts the cytotoxic effect of anandamide. Abbreviations used: AEA: anandamide; CB1: cannabinoid receptor type 1; FAAH: fatty acid amide hydrolase; NAPE-PLD: N-acyl phosphatidylethanolamine-specific phospholipase D.

Figure 3 shows the changes in expression of CB1 receptor, FAAH, and NAPE-PLD enzymes during preimplantation embryo development and migration to the endometrial cavity.

Based on the available evidence, it can be concluded that eCB signaling definitely holds potential as a therapeutic target in multiple female reproductive events. CB1, NAPE-PLD, and FAAH could be key regulators of reproduction, so we can assume that drugs capable of modulating their activity could influence fertility rates. In particular, CB1 is almost ubiquitous; it appears difficult to make it a therapeutic target. In the case of NAPE-PLD, inhibitors of this enzyme could be useful in treating or preventing implantation defects in relation to its role in controlling AEA concentrations. Finally, FAAH seems the most ideal target for drug development in combating infertility [60].

In conclusion, an emerging concept is that the biological activity of eCBs also depends on their intracellular trafficking by distinct carriers, collectively called eCB intracellular transporters (EITs) [43], so it appears crucial to understand how EITs can drive the same eCB to trigger different signaling pathways in different cellular contexts. Concerning this concept, recent evidence based on pharmacological and genetic manipulation has demonstrated that different EITs may drive AEA signaling at different receptors and/or AEA to be metabolized by different enzymes [44]. Thus, compounds selectively directed at one or more of these novel EITs could lead to the development of intensely searched ECS-based therapeutics with limited side effects and abuse liability.

### Epigenetic Mechanisms and Female Gametogenesis

The mechanism by which cannabis exposure alters female fertility is yet to be determined. A recent study focused on two possible mechanisms: the first, related to the disruption of the endocannabinoid system, and the second with the possible epigenetic modifications caused by exposure to phytocannabinoids. A significantly higher expression of CB2 than of CB1 was observed in human granulosa cells both in vivo and in vitro. In particular, despite the fact that the expression levels of both CB1 and CB2 receptors in the surrounding granulosa cells were not affected by the presence of phytocannabinoids in vivo, a significant increase in CB2 expression was observed in vitro after a combined treatment with Δ9-THC and its two main metabolites (11-OH and 11-COOH-THC) [61].

As previously discussed, cannabis exposure—like other environmental factors—can induce epigenetic alterations. It is well documented that Δ9-tetrahydrocannabinol (Δ9-THC) can lead to widespread histone modifications and changes in DNA methylation patterns across various tissues, including the brain, sperm, blood cells, and ovarian follicles. Nevertheless, there remains a considerable gap in our understanding of how cannabis influences the epigenetic landscape of somatic cells involved in oocyte development and maturation. Whether phytocannabinoids interfere with the physiological roles of endocannabinoids in reproductive function is still an open question. However, emerging evidence from both animal studies and investigations in human granulosa cells indicates that phytocannabinoids are indeed capable of eliciting epigenetic changes [61].

Regarding the epigenetic modifications as a possible mechanism for the effect that phytocannabinoids (PCs) exert on female fertility, it a significantly reduced expression of the de novo methylating enzyme DNMT3b in follicular fluid samples from patients who had consumed cannabis was observed. These findings were further validated in vitro. These changes were demonstrated by treating granulosa cells (GCs) either with Δ9-THC alone or by treating GCs with a combined treatment of all three metabolites, mimicking a real-life scenario following cannabis consumption. The observed effect was substantial, dose-dependent and cumulative up to 120 h of treatment [61].

In exploring epigenetic modifications as a potential mechanism through which phytocannabinoids (PCs) impact female fertility, a significant downregulation of the de novo DNA methyltransferase DNMT3b was identified in follicular fluid samples from individuals with a history of cannabis use. These observations were corroborated through in vitro experiments, where granulosa cells (GCs) were exposed either to Δ9-THC alone or to a combination of its primary metabolites, simulating more accurately the physiological conditions following cannabis intake. The resulting epigenetic changes were both robust and dose-dependent, with cumulative effects observed over a 120-h treatment period [61].

The process of germ-cell development is regulated by both genetic and epigenetic mechanisms. Cannabinoids can cross the placenta, enter the fetal bloodstream, and distribute to fetal tissues causing several adverse outcomes; however, the impact on germline development and the long-term effects on the reproductive functions of the offspring are still not completely known [62]. Recently it was investigated the effects of prenatal exposure to a selective CB2 agonist, JWH-133, on the long-term reproductive health of male and female human offspring and on the involved molecular epigenetic mechanisms. It has been proven that prenatal activation of CB2 has a sex-specific impact on germ cell development of the offspring. While in males, it determines a delay of germ cell differentiation coinciding with an enrichment of H3K27me3, in females, it causes a reduction of the follicle number through an increased apoptotic process not linked to modified H3K27me3 level [42,62].

In summary, this study highlights the long-term effects of CB2 overactivation in fetal male and female germ cells and indicates that altered CB2 signaling impairs male and female germ cell development through different mechanisms.

Considering that in humans a low ovarian reserve is an important cause of infertility, the knowledge of the potential long-term consequences of cannabinoid exposure during pregnancy on the reproductive health of the offspring may increase the risk perception of cannabis use in pregnant women [39].

## 4. Conclusions

In conclusion, understanding the prevalence and impact of male and female infertility is essential for developing effective interventions and mitigating risk factors and consequences on reproductive health. Recent studies have highlighted the crucial role of the endocannabinoid system (ECS) in the complex regulatory processes governing both male and female reproduction. Investigating the mechanistic involvement of the ECS in fertility could provide valuable insights into the pathophysiology of infertility.

By characterizing the expression and function of cannabinoid receptors and endocannabinoids in sperm cells and oocytes, a deeper understanding of their specific roles in reproductive function can be achieved. Moreover, exploring ECS signaling pathways and their interactions with other regulatory systems, such as hormonal networks and oxidative stress responses, may contribute to a more comprehensive model of fertility regulation.

Additionally, with the progressive decriminalization and increasing use of marijuana, concerns regarding the impact of exogenous cannabinoids, particularly THC and CBD, have grown on fertility. Notably, THC and CBD, much like their endogenous counterparts, can disrupt ECS function, potentially impairing fertility at multiple levels. In females, these disruptions may affect embryo oviductal transport, implantation, and fetal development, while in males, they can alter sperm survival, motility, capacitation, and the acrosome reaction.

Therefore, clinicians, particularly ART specialists, should routinely assess patients’ marijuana consumption when evaluating fertility and provide comprehensive counseling on the potential reproductive risks of cannabinoid use. Furthermore, health professionals should carefully consider the association between marijuana exposure and reproductive outcomes when prescribing medical cannabis, ensuring that patients are fully informed about its possible implications for fertility.

The identification of the ECS as a novel biomarker family for enhancing diagnostic accuracy in infertility assessment presents significant potential for clinical application. While current research underscores the critical role of the ECS in fertility, further investigations are needed to fully elucidate its underlying mechanisms and broader implications for reproductive health.

Lastly, understanding the epigenetic modifications associated with the ECS may have profound implications for health maintenance and disease prevention. This suggests that targeted modulation of the ECS could represent a promising therapeutic approach not only for reproductive disorders, but also for long-term transgenerational health benefits in non-exposed future generations.

## Abbreviations

The following abbreviations are used in this manuscript:

2-AG	2-arachidonyl-glycerol
ABHD2	abhydrolase domain-containing protein 2
AEA	anandamide or N-arachidonoylethanolamide
ART	assisted reproductive technology
cAMP	cyclic adenosine monophosphate
CB1	cannabinoid receptor type 1
CB2	cannabinoid receptor type 2
CBD	cannabidiol
CNR1	cannabinoid receptor type 1 (gene)
CNS	central nervous system
eCB	endocannabinoid
ECS	endocannabinoid system
ECSS	endogenous cannabinoid signaling system
EITs	endocannabinoids intracellular transporters
EMT	epithelial-to-mesenchymal transition
ERE	estrogen-responsive element
ERβ	estrogen receptor beta
FAAH	fatty acid amid hydrolase
FSH	follicle-stimulating hormone
GPCR	G-protein-coupled receptor
GV	germinal vescicle
GCs	granulosa cells
H3K9	histone H3 lysine 9
hGCs	human granulosa cells
ICSI	intracytoplasmatic sperm injection
JWH-133	3-(1′1′dimethylbutyl) 1-deoxy-8-THC
LSD1	lysine-specific demethylase 1
MI	meiosis I
MII	meiosis II
MGLL	monoacylglycerol lipase
miRNAs	microRNAs
NAPE-PLD	N-acyl phosphatidylethanolamine phospholipase D
OEA	oleoylethanolamide
PCE	prenatal cannabis exposure
PCs	phytocannabinoids
PEA	palmytoylethanolamide
PGCs	primordial germ cells
PRM1	protamine 1
PRM2	protamine 2
ROS	reactive oxygen species
SSCs	spermatogonial stem cells
THC	∆9tethrahydrocannabinol
TP1	transition protein 1
TP2	transition protein 2
TRPV1	transient receptor potential cation channel subfamily V member 1
